# Agranulocytosis with tonsillitis associated with methimazole therapy

**DOI:** 10.1016/S1808-8694(15)31339-2

**Published:** 2015-10-20

**Authors:** Jorge T. Zambrana, Fábio F.T. Zambrana, Firmino R.S. Neto, André L.C. Gonçalves, Fernanda F. . Zambrana, Jorge Ushirohira

**Affiliations:** ^1^ Faculty Professor, Discipline of Otorhinolaryngology, Medical School, Itajubá; ^2^ Joint Professor, Discipline of Otorhinolaryngology, Medical School, Itajubá; ^3^ Former intern, Service of Otorhinolaryngology, University Hospital, Medical School Itajubá; ^4^ Resident physician, Service of Otorhinolaryngology, University Hospital, Medical School Itajubá; ^5^ Resident physician, Service of Otorhinolaryngology, University Hospital, Medical School Itajubá; ^6^ Resident physician, Service of Otorhinolaryngology, University Hospital, Medical School Itajubá; Study carried out at University Hospital, Medical School Itajubá

**Keywords:** hyperthyroidism, methimazole, agranulocytosis, tonsillitis

## Abstract

**T**he treatment of hyperthyroidism with antithyroid drugs can cause a significant side effect in 0.2 to 0.3% of the cases: agranulocytosis. Infectious complications caused by this condition affect mainly the throat, and tonsillitis is one of its manifestations. The present study reported the case of a female patient, 33 years old, manifesting odynophagia and fever resistant to many antibiotics. The patient showed hyperthyroidism and had been using methimazole for two months. With the diagnoses of agranulocytic angina, the drug was withdrawn and treatment with ciprofloxacin, symptomatic drugs and granulocytic-colony stimulator, besides fluconazol was started. The patient developed satisfactorily, being discharged ten days after the beginning of the treatment. Fifteen days later, total thyroidectomy was carried out. The purpose of this report was to point out the importance of knowing the side effects of drugs to advise the patients about them and to warn physicians about the need for evaluating the patient as a whole, searching for other current diseases and drugs in use.

## INTRODUCTION

Hyperthyroidism consists of an increase in synthesis and release of thyroid hormones and thyrotoxicosis is the resulting clinical syndrome^1^. It is a common disease, with predominance of 0.2 to 0.5% in the population^2^. The most frequent cause of thyrotoxicosis is Basedow-Graves disease or toxic diffuse goiter, responsible for 60 to 90% of the cases, and observed in women at a frequency of 1.9%, with a peak between the ages of 20 and 40 anos^1^. It is an autoimmune disease with family tendency and association with other endocrine autoimmune diseases (IDDM and Adisson) and non-endocrine diseases (pernicious anemia, myasthenia, rheumatoid arthritis, Sjögren disease, vitiligo and chronic active hepatitis)^3^.

Regardless of the cause factor, signs and symptoms resulting from thyrotoxicosis are: nervousness, emotional instability, fatigue, excessive sudoresis, weight loss, tremors, tachycardia, ocular complaints (GRAVES disease) and goiter. Some environmental factors trigger an immune response in Graves such as: pregnancy, iodine excess, lithium therapy, renal infections (since they induce expression of DR4 in follicular cells) or bacterial infections (*Yersinia enterocolitica* has cross reaction with thyroid antigens) and use of corticosteroids^5^.

The increase in thyroid size, infiltration, ophthalmopathy and dermopathy are mediated by autoimmune processes, differentiating Graves disease from other causes of thyrotoxicosis^1^.

There are three main treatment approaches: anti-thyroid drugs, surgery and radioiodine, but initial therapy normally prefers pharmacological treatment, frequently methimazole4., 6.. It is a drug derived from thyourea or thyonamide that inhibits oxidation of iodine captured by the thyroid and as a consequence, it prevents synthesis of thyroid hormones^7^.

The less uncommon side effect of the use of thyourea, even though very important, is agranulocytosis^1^. It may be present regardless of age, dose and treatment duration^8^, even though Meyer-Gebner et al. have reported higher incidence with higher doses^6^. It has sudden onset and affects 0.2 to 0.3% of the patients8., 9.. Tamai et al., after a review of 7,000 patients with diagnosis of Basedow-Graves, found agranulocytosis in only 12 patients with incidence of 0.17%^8^. To Meyer-Gebner et al. The incidence found in 1,256 studied patients was of 0.18%^6^.

Based on this side effect, patients need to be instructed to stop medication and get in contact with their physician in case of fever or infection, especially of the oropharynx, considering that the clinical evidence of infections associated with the use of anti-thyroid drugs is usually symptomatic in this region1., 8., 9.. Periodontal, periapical and oral mucosa infections tend to aggravate quite quickly and they are the main foci of infection, and gram negative are the most common agents^10^. Monitoring of patients by routine counting of leukocytes has been proposed by same authors as effective in the prevention of agranulocytosis^11^. However, there is no literature consensus and according to Meyer-Gebner et al. it would be important to have appropriate patient guidance for them to identify the symptoms of the disease^6^.

Agranulocytic angina ranges from simple erythema to ulceration and necrosis of buccopharyngeal mucosa^12^. There is no tendency to hemorrhage, nor generalized lymphadenopathy and splenomegalia, such as in acute leukemia^12^. Complete blood count shows marked leukocytopenia: 2,000, 1,000, 500 leukocytes/mm^3^, together with neutropenia, which may be of 0%^12^. There are no abnormal or immature leukocytes. Red blood cells and platelets are not affected^12^. The identification of agranulocytosis using complete blood count requires management of wide-spectrum antibiotics, especially against *Pseudomonas aeruginosa*, as well as support therapy. Currently, agranulocytosis induced by methimazole is a reversible process9., 10..

The purpose of the present study was to present an uncommon case of agranulocytic angina caused by use of methimazole and to warn physicians about the need to consider this diagnosis in patients that have taken this medication. It is especially recommended that these patients be informed about the possible drug side effects, a practice that may prevent suffering and unnecessary costs.

## CASE REPORT

Female patient aged 33 years, came to the outpatient unit of Otorhinolaryngology, University Hospital, Medical School, Itajubá (MG), with complaint of odynophagia and fever for one month. In the anamnesis, she showed to be taken aback and anxious with the persistence of her complaints, even after the use of different drugs, as confirmed by a number of prescriptions she had: penicillin benzatin, sulfamethoxazole + trimetoprim, amoxicillin, lincomycin and ceftriaxone.

During patient's examination, we detected marked exophthalmia associated with the presence of goiter. When asked about it, she informed that for 2 months she had started clinical treatment fort hyperthyroidism with methimazole 80mg/day. She informed that the physician had not instructed her to interrupt treatment in case of fever or sore throat.

**Figure 1:**
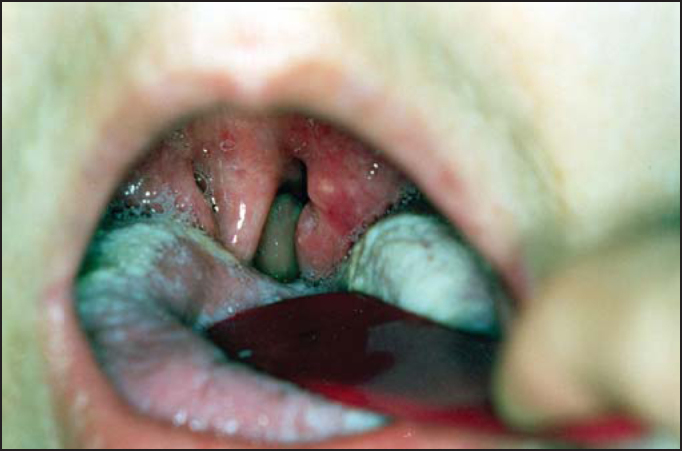
Oropharyngoscopy showing erythematous aspect of palatine tonsils, uvula edema and tongue monolyasis.

**Figure 2:**
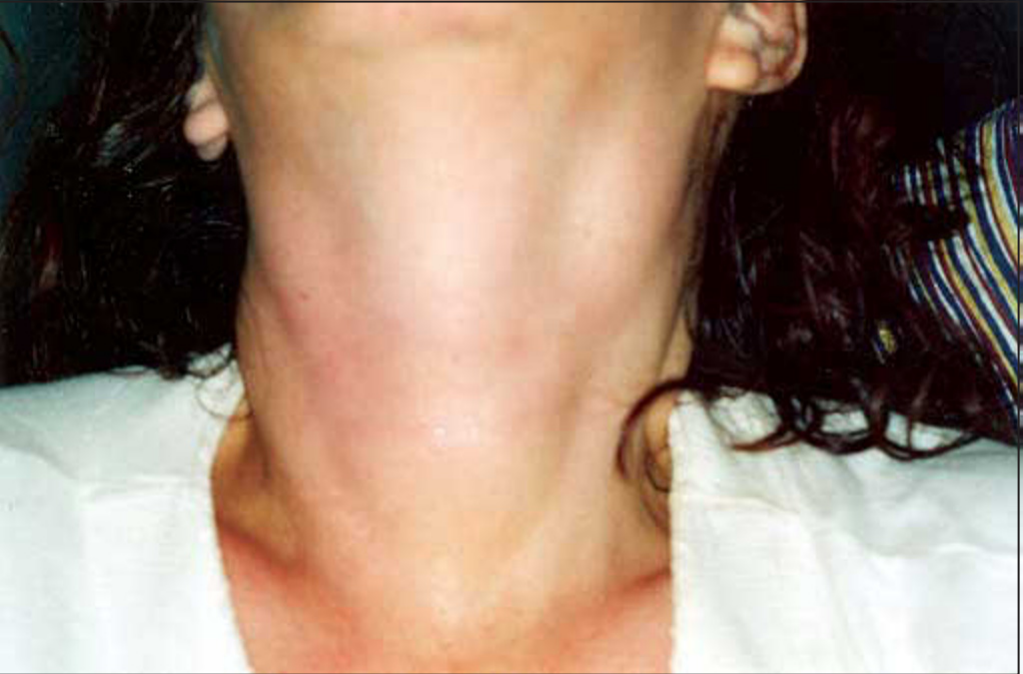
Neck inspection: note the goiter and lateral chain lymphoadenomegaly.

ENT physical examination showed positive data for the presence of erythematous tonsillitis and cervical lymphadenomegalia (Figures 1 and 2). The general examination showed tachycardia and tachypnea. She was febrile and hypotense. Based on the diagnostic hypothesis of agranulocytic angina secondary to methimazole, we requested a complete blood count that showed significant leukopenia: 1,300/mm^3^. For the differential diagnosis, we also requested serology for infectious mononucleosis and AIDS, which were negative.

We decided to hospitalize the patient and to jointly follow her up with the clinical practice medicine in the hospital. We ordered a myelogram to exclude the presence of some possible myeloproliferative diseases, excluded after the result. The treatment was performed by suspending methimazole, administering symptomatic and vasopressure drugs, intravenous antibiotic therapy with ciprofloxacin and granulocytic colony stimulating agent.

**Figure 3:**
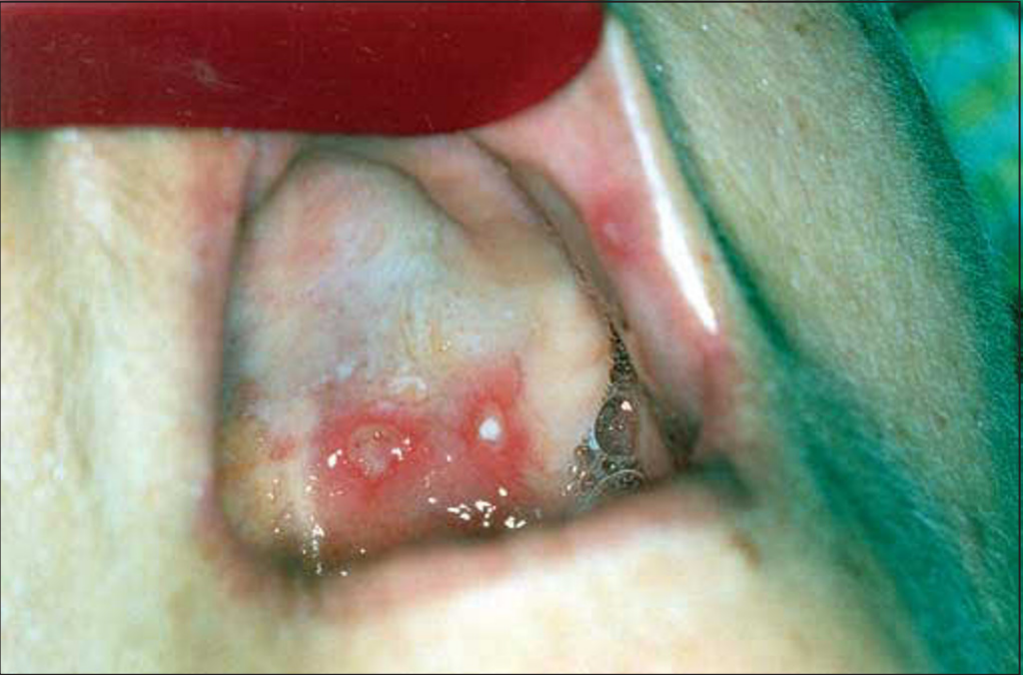
Aphthous lesions affecting the hard palate and jugal mucosa.

During hospitalization, she presented affection of the oropharynx with diffuse apthae and monolyasis (Figure 3), when we started treatment with fluconazole 300 mg/day. She presented satisfactory evolution, with progressive increase of leukocyte count and remission of oral lesions, being discharged with good general status after 10 days of hospital stay. Fifteen days after the discharge, she was submitted to total thyroidectomy for definitive cure of thyrotoxicosis and currently she is followed up as an outpatient by endocrinology.

## DISCUSSION

The case reported here is of a patient who came to the Service of Otorhinolaryngology and whose main complaint was odynophagia and fever; she had been submitted to different antimicrobial treatment regimens, with different spectrum coverage, all of them without any efficacy. At that time, it was essential to collect a detailed history, especially concerning other systemic diseases that could interfere with the immune system of the patient and recent treatment of other pathologies. In view of the fact that she had been using methimazole for the treatment of hyperthyroidism, associated with exophthalmia and goiter, we suspected of a case of tonsillitis with modification of leukocyte count, a suspicion confirmed by the leukocyte count test.

According to the studied literature, agranulocytosis is a very infrequent side effect in patients treated with methimazole1., 6., 8., 9.. On average, the onset of this complication is present in the first weeks of treatment with the referred drug^6^. In the present study, the patient had been taking the drug for 2 months.

According to the literature, antibiotics, corticoids and granulocytic colony stimulators are the most frequently used drugs to treat agranulocytosis2., 6., 9.. In the reported case, the patient improved her manifestations by symptomatic medication, fluconazole, ciprofloxacin and granulocytic colony stimulator. Moreover, the patient was followed up also by the general clinical care of the institution.

This report shows the importance of considering the patient as a whole, trying to associate signs and symptoms to a possible systemic impairment caused by other diseases or as a side effect of some drug in use. It is important to make physicians aware of the need to get to know the most severe side effects and to inform patients about the situations that may take place during treatment and that can pose a risk to their lives.
